# Ionic Liquid Coating-Driven Nanoparticle Delivery to the Brain: Applications for NeuroHIV

**DOI:** 10.21203/rs.3.rs-2574352/v1

**Published:** 2023-02-14

**Authors:** Christine M. Hamadani, Fakhri Mahdi, Anya Merrell, Jack Flanders, Ruofan Cao, Priyavrat Vashisth, Mercedes C. Pride, Alysha N. Hunter, Gagandeep Singh, Gregg Roman, Jason J. Paris, Eden E. L. Tanner

**Affiliations:** University of Mississippi; University of Mississippi; University of Mississippi; University of Mississippi; University of Mississippi; University of Mississippi; University of Mississippi; University of Mississippi; University of Mississippi; University of Mississippi; University of Mississippi; University of Mississippi

## Abstract

Delivering cargo to the central nervous system (CNS) remains a pharmacological challenge. For infectious diseases such as HIV, the CNS acts as a latent reservoir that is inadequately managed by systemic antiretrovirals (ARTs). ARTs thus cannot eradicate HIV, and given CNS infection, patients experience an array of neurological deficits that are collectively referred to as ‘neuroHIV’. Herein we report the development of bioinspired ionic liquid-coated nanoparticles (IL-NPs) for *in situ* hitchhiking on red blood cells (RBCs), which enabled 48% delivery of intravenously infused cargo to the brain. Moreover, the ionic liquid (IL) choline trans-2-hexenoate (CA2HA 1:2) demonstrated preferential accumulation in parenchymal microglia over endothelial cells post-delivery. We further demonstrate the successful loading of abacavir (ABC), an ART that is challenging to encapsulate, into the IL-coated NPs and verify the retention of antiviral efficacy *in vitro*. IL-NPs were not cytotoxic to primary human peripheral blood mononuclear cells (PBMCs) and the CA2HA 1:2 coating conferred notable anti-viremic capacity on its own. In addition, *in vitro* cell culture assays showed markedly increased uptake of IL-coated nanoparticles into neuronal cells compared to bare nanoparticles. This work debuts bioinspired ionic liquids as promising nanoparticle coatings to assist CNS biodistribution and has the potential to revolutionize the delivery of cargos (i.e., drugs, viral vectors) through compartmental barriers such as the blood-brain-barrier (BBB), illustrated in the graphical abstract below.

## Drug delivery barriers limiting antiretroviral (ART) therapy to the brain may be overcome via red blood cell (RBC) hitchhiking in situ

HIV is present in the CNS soon after infection and establishes a reservoir of latently-infected microglia, the long-lived immune cells that reside therein^1–5^. Astrocytes are also infected, albeit in much lesser proportion and their capacity for replication-competent activation is controversial^6–9^. This reservoir, and others throughout the body, are not effectively treated by ART^10,11^. In the CNS, ART is impeded by BBB efflux^12,13^ and preferentially accumulates in off-target cell types (e.g. endothelial cells^14^), reducing the capacity for therapeutic concentrations to be achieved in cells comprising the central reservoir, such as microglia^15,16^. Overcoming viral latency has posed an additional challenge that has found some recourse in novel strategies of latency reversing agents (LRAs) and genome-editing techniques that may eradicate HIV^17–20^. However, even if successful, these compounds and constructs need to be targeted to HIV reservoirs which remains to be achieved.

To overcome these challenges, we developed an ionic liquid-coated nanoparticle (IL-NP) as a delivery vehicle to transport ART to the brain. Ionic liquids (ILs) are composed of bulky, asymmetric anions and cations and are liquid < 100°C^21^. When they are synthesized from bioinspired components, they retain high biocompatibility and have been used in a variety of drug delivery applications, including transdermal, buccal, subcutaneous, and oral delivery of therapeutics^22–25^. We have previously shown that choline trans-2-hexenoate (CA2HA 1:2) can be used to coat polymeric nanoparticles, and when mixed with whole blood, show spontaneous attachment (“hitchhiking”) onto red blood cells. When intravenously (IV) injected into the tail vein, 46.6 ± 13.5% (n = 6, ± standard deviation) of the delivered dose accumulated in the lungs, the first encountered capillary bed from systemic circulation^26,27^. Based on past work by Muzykantov et al^28–30^, we hypothesized that IV injection into the carotid artery would instead confer similar rates of brain accumulation, due to RBC shearing behavior through the BBB.

## Nanoformulation & Characterization Of Il-plga Nps For Brain Delivery Of Arts Via Intravenous (Iv) Administration

We first prepared carboxylic acid-terminated poly(lactic-co-glycolic) acid (PLGA)-based nanoparticles (NPs) by nanoprecipitation and solvent evaporation of acetonitrile (ACN) as previously described^26^ and as detailed in the Methods section. This produced bare, unloaded particles of 45.1 ± 4.8 nm hydrodynamic diameter, −26.6 ± 5.2 mV surface charge, and 0.17 ± 0.06 polydispersity index (PDI) by Dynamic Light Scattering (DLS, n = 4).^46^ We then loaded our particles with either equivalent amounts of abacavir (ABC) or far-red fluorescent dye 1,1′-dioctadecyl-3,3,3′,3′- tetramethylindodicarbocyanine, 4-chlorobenzenesulfonate (DiD) at approximately 2% by weight of the polymer (normalized to cargo molecular weight) into the organic phase. ABC-loaded NPs (n = 7) had diameters of 76.4 ± 13.5 nm, had a stable surface charge of −36.9 ± 9.8 mV, and a monodisperse PDI of 0.09 ± 0.04, while the DiD-loaded bare NPs (n = 3) were 61.6 ± 1.3 nm, −24.8 ± 0.26 mV, and 0.11 ± 0.015 PDI, indicating slightly improved loading abilities for ABC over DiD.

**Figure 1:**
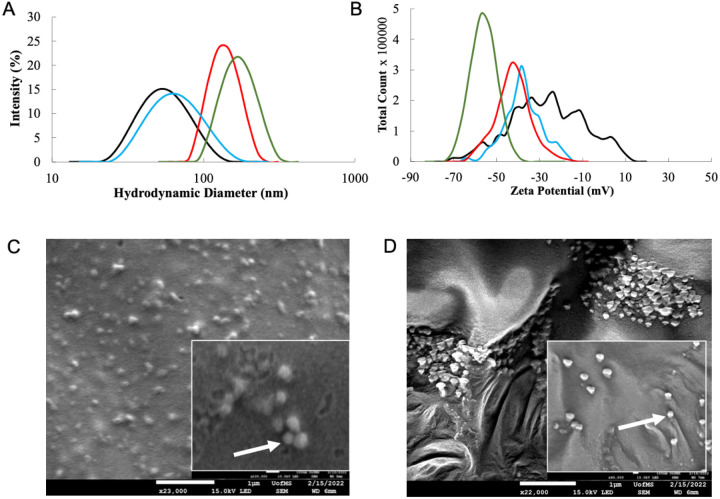
Ionic liquid coats both empty PLGA NPs and those loaded with 60 ug/mL abacavir. Empty bare PLGA (black line) and IL-coated PLGA (red line) NPs undergo an increase in size (A) and anionic shift in surface charge (B) when abacavir is loaded into Bare PLGA (blue line) and IL-coated PLGA (green line) NPs. Scanning Electron Microscopy (SEM) of Bare PLGA (C) and IL-coated PLGA (D) shows morphological changes upon IL coating. Scale = 1 μm.

The loaded bare NPs were then coated with choline 2-hexenoate (CA2HA 1:2) IL (IL-PLGA NPs) by placing a single ~ 10 mg liquid drop in the center of the vial (10 mg neat IL/mg PLGA), and were stirred for 2 more hours. In each case, the previously bare NPs increased in size, and decreased in surface charge while maintaining a monodisperse PDI below 0.2. ABC-loaded IL-coated NPs were 191.5 ± 23.6 nm, −54.8 ± 6.5 mV, and had a PDI of 0.12 ± 0.07 (n = 5). Figure 1 shows the size (A) and surface charge (B) of the bare empty PLGA (black), ABC-loaded PLGA (blue), IL-coated empty PLGA (red), and ABC-loaded IL-coated PLGA (green), as well as C) bare and D) IL-coated NP morphology by Scanning Electron Microscopy (SEM). Full DLS data is detailed in Table S1.

The encapsulation efficiency (EE) of DiD was determined by fluorescent plate reader (Fig. S2) (DiD, 2% (v/v): 60.43 ± 2.03% (n = 3)), while the presence of ABC was measured by quantitative ^1^H NMR spectroscopy (Fig. S3) (ABC, 2% (v/v), estimated ~ 62.7 ug/mL from an added 74.1 ug/mL, or ~ 84.6% EE). The quantitative difference in encapsulation efficiency discovered between DiD and ABC is consistent with the DLS findings, as shown below in Fig. 1.

### ART-Encapsulated IL-PLGA NPs suppress HIV viral replication, enhance cellular uptake of, & are biocompatible to, human peripheral blood mononuclear cells (PBMCs)

**Figure 2:**
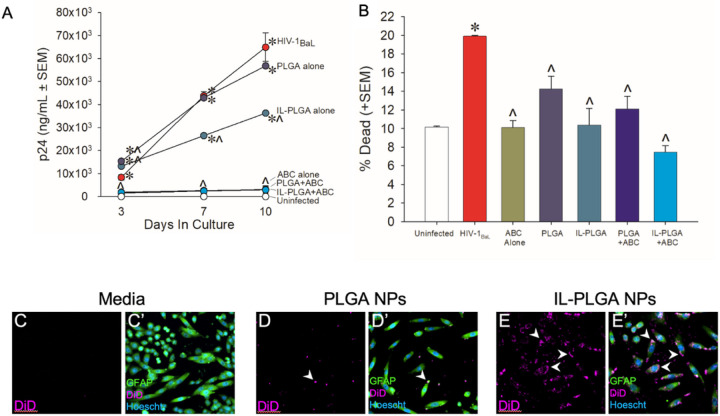
IL-coated PLGA NPs encapsulate abacavir, suppress viral replication in HIV-1 treated human PBMCs without cytotoxicity, and show enhanced human microglia and human astrocyte uptake *in vitro*. (A) HIV-1_BaL_ viral replication (n=2) is attenuated by CA2HA 1:2-coated PLGA NPs loaded with abacavir (ABC; 60 μg/mL; n=3), ABC alone (n=3), or CA2HA 1:2-coated empty PLGA NPs (n=3). *indicates significant difference from mock-infected cells (n=2); îndicates significant difference from HIV-infected cells; *p* < 0.05 (Repeated-Measures ANOVA). (B) Bare and coated PLGA NPs with ABC (60 ug/mL) show little cytotoxicity when compared to mock-infected PBMCs. *indicates significant difference from mock-infected cells; *indicates significant difference from mock-infected cells; îndicates significant difference from HIV-1 infected cells; *p* < 0.05 (Repeated Measures ANOVA). (C-E’) Immunocytochemistry on co-cultured primary human astrocytes and primary human microglia were performed: (C-C’) media-control, (D-D’) PLGA NPs loaded with DiD (purple), and (E-E’) IL-PLGA NPs loaded with DiD. Cells were co-labeled with anti-Iba-1 or -GFAP (green) and Hoechst nuclear stain (blue). Intracellular DiD accumulation was qualitatively greater when PLGA-NPs were coated with IL (see E-E’).

To assess the bioactivity of ART when encapsulated inside PLGA & IL-PLGA NPs, HIV-1 replication was assessed in human peripheral blood mononuclear cells (PBMCs) that were mock-infected or were infected with HIV-1_BaL_ (1 ng/mL) for 10 days. PBMCs were treated with 1 mg/mL bare or IL-coated PLGA NPs that were either unloaded or loaded with abacavir (ABC, 60 μg/mL). ABC was also administered alone as a control. Concentration of the HIV-1 capsid protein (p24, ng/mL) was assessed on days 3-, 7-, and 10 post-infection by enzyme-linked immunosorbent assay (ELISA) (Fig. 2A). As expected, viral replication was significantly greater in cells treated with HIV-1 alone, empty NPs, or empty IL-coated NPs compared to those that were mock-infected (*p* < 0.0001–0.0009). Compared to HIV-infected cells, ABC significantly attenuated viral replication when administered alone and retained its bioactivity when encapsulated in NPs (*p* < 0.0001). Intriguingly, IL-coated NPs significantly attenuated HIV-1 replication on their own (*p* = 0.04); while ILs been have previously demonstrated to exert virucidal effects^33–37^, this has not been previously demonstrated with HIV-1.

PBMC viability was also assessed at the 10-day timepoint via a trypan blue exclusion assay (Fig. 2B). As expected, HIV-1 significantly increased the proportion of dead cells (*p* = 0.0004)^38^; any other treatment significantly attenuated this (*p* = 0.0002–0.02). Additionally, when visualized by fluorescent microscopy, the uptake of DiD far-red fluorescent dye in co-cultured human astrocytes and human microglia cells (Fig. 2C – E’) was dramatically enhanced when carried by IL-PLGA NPs (Fig. 2E and E’) compared to bare PLGA NPs (Fig. 2D and D’) or media alone (Fig. 2C and C’). This increased uptake could be partially explained by lipid extraction behavior engaged by the choline hexenoate coating, which has been shown before to increase uptake in RAW 264.7 macrophages^27^ or the carboxylic anion interacting with the MCT1 proteins present on the astrocytic cell surfaces^43–45^.

### Carotid IV injection directs IL-PLGA NPs to the brain and results in regional Abacavir (ABC) brain accumulation

To test the *in vivo* delivery efficacy, DiD-loaded bare and IL-coated PLGA NPs were intravenously (IV) injected into the carotid artery (500 μL) of healthy, 8-week-old, female, Sprague Dawley rats with in-dwelling carotid catheters (n = 4/group). At 6 hrs, the rats were sacrificed and exsanguinated. Blood was collected by cardiac puncture and immediately analyzed by Fluorescence Activated Cell Sorting (FACS) (Fig. S4). Blood-filtering organs were subsequently harvested (brain, lung, heart, liver, spleen, and kidneys) (n = 3/group). From each treatment group, one animal underwent transcardial perfusion with phosphate buffer saline (1x PBS pH 7.4) followed by 4% PFA. One fixed brain from each group was used for epifluorescent imaging while the other three sets of organs were flash frozen and stored at −80°C to perform biodistribution analysis. No physiologically-adverse effects of NPs were observed during live study or post-mortem.

**Figure 3:**
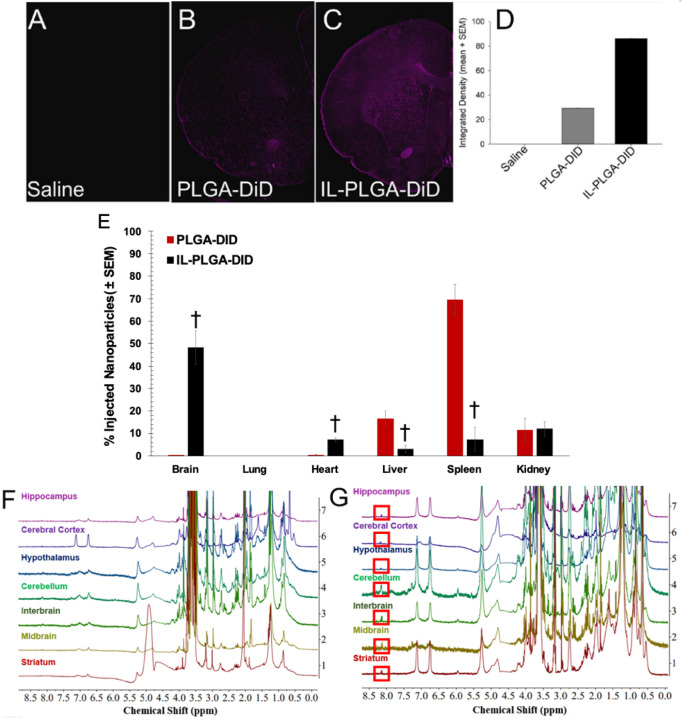
IL-NPs dramatically enhance delivery to the brain *in vivo* and influence regional abacavir accumulation. (A-D) Sprague-Dawley rat brain cross-sections shown after treatment with: (A) saline, (B) bare NPs loaded with DiD (purple), or (C) IL-NPs loaded with DiD. (D) Signal quantified by densitometry (area × mean intensity; n=1/group). (E) Biodistribution (%) of injected DiD in isolated organs (% ID organ, n=3/group; mean + SEM). †denotes significant difference from respective PLGA-DiD-treated group; *p* < 0.05 (two-tailed Student’s *t*-test). (F-G) Representative differences, by ^1^H-NMR spectroscopy, in abacavir (ABC) regional brain accumulation in Sprague-Dawley rat brains (n=3/group) post intra-carotid injection for (F) empty IL-PLGA NPs and (G) IL-PLGA NPs loaded with ABC. Key proton peak for ABC presence at 8.1 ppm is indicated (see red box, panel G).

As shown in Fig. 3, notable differences in raw DiD signal were observed for IL-coated vs. uncoated DiD NPs in the brain via wide-field epifluorescence images (Fig. 3A–C). Compared to saline-infused rats (Fig. 3A), a faint DiD signal was detected in those infused with DiD-loaded NPs (Fig. 3B) compared to a much more intense signal for rats infused with IL-coated NPs (Fig. 3C; densitometric quantification in Fig. 3D). Figure 3E shows the results of the quantitative biodistribution study (n = 3/group, ± SEM). Bare PLGA NPs accumulated primarily in the spleen (69.6 ± 6.9%), with a smaller amount in the liver (16.6 ± 3.3%), kidneys (11.6 ± 5.2%), and the least in the brain (0.1 ± 0.1%). In contrast, the IL-coated NPs demonstrated the greatest accumulation in the brain (48.1 ± 7.5%), with lesser concentration in the kidneys (12.1 ± 3.2%), heart (7.3 ± 0.8%), spleen (7.3 ± 5.5%), and least in the liver (3.02 ± 1.6%). However, there appeared to be no detectable accumulation in the lungs post-intracarotid injection. This finding contrasts with earlier work carried out via tail vein injection, suggesting that the target organ is critically dependent on the site of IV injection, which is consistent with prior RBC hitchhiking reports^28–30^. The major innovation reported herein reveals the capacity for IL–NPs to hitchhike onto the RBCs post-injection, while earlier work required removal of the RBCs and NP attachment *ex vivo*^28–31^. This is possible given that the IL coating imbues stealth properties onto the NP, allowing it to navigate the plasma and serum proteins to contact other blood components, even outperforming poly(ethylene) glycol^26^.

Once biodistribution was determined with DiD, a new set of healthy, 8-week-old, female, Sprague Dawley rats with in-dwelling carotid catheters received IV infusions (under the same conditions) with either empty, or ABC-encapsulated, IL-coated PLGA NPs (n = 3/group). Sites of regional distribution of abacavir cargo were evaluated within the brain (at the same 6-hour endpoint). Brain subregions (i.e., cerebral cortex, hippocampus, striatum, hypothalamus, midbrain, cerebellum, and interbrain) were grossly dissected, subsequently homogenized, and processed for assay via ^1^H-NMR spectroscopy to identify areas of selective ABC accumulation. As illustrated by both Fig. 3F and 3G, a broad range of proton peaks were found in the filtrate corresponding to those of the IL-PLGA NPs (such as the methyl or CH_2_ group off the anion alkyl chain at 0.9 ppm and 1.5 ppm, or the protons off the trans-2-double bond between 6.5–7.5 ppm), albeit shifted due to the NP degradation process during extraction (when compared to intact IL-NPs in Fig. S3 C&E). While also slightly shifted due to the co-solvent composition during extraction, Abacavir’s signature singlet proton peak is clearly distinguishable from the baseline at 8.1 ppm (Fig. S3 F&G) in Fig. 3G, when compared to the empty cargo IL-NP delivery (Fig. 3F). Abacavir was observed to accumulate most greatly in the cerebellum, interbrain, striatum, and midbrain regions, with lesser (but considerable) delivery to the hippocampus, cerebral cortex, and hypothalamus regions (Fig. 3G). It seems likely that the intracarotid path of microvascular distribution contributed to this pattern of particulate accumulation, with IL- PLGA NPs shearing off from RBCs and subsequently crossing the BBB^40,41^. Interestingly, as microglial populations are vastly diverse throughout these brain subregions, the potential for deep and comprehensive microglial targeting during HIV can be possible with such a distribution^42^.

### IL-PLGA NPs enter the brain by shearing through blood vessels and traffic to microglia for selective uptake

**Figure 4:**
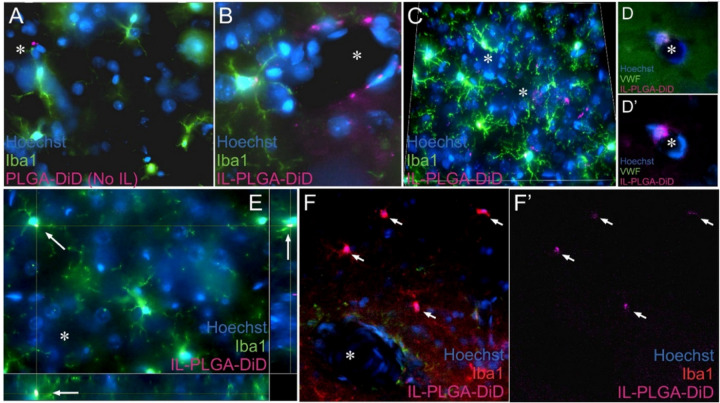
IL-PLGA NPs enter the brain by shearing through blood vessels and are uptaken by microglia in the caudate/putamen. (A) Bare PLGA NPs were sporadically seen in endothelial cells. (B,C) IL-PLGA NPs were seen in parenchymal microglia and around blood vessels in endothelial cells. (C) DiD signal from IL-coated NPs is observed in every microglial soma captured in the field and in several suspected endothelial cells surrounding apparent blood vessels. (D,D’) Labeling with von Willebrand factor confirmed the presence of DiD in endothelial cells. (E) Z-stack imaging supports intracellular localization of DiD in microglia (see bottom and right orthogonal views for virtual cross-section). (F,F’) DiD colocalization to microglia was frequently uniform next to large vessels. *Indicates blood vessel. Arrows localize DiD in panels E, F, and F’. Images collected using a 63x/NA1.4 oil lens.

Bare PLGA NPs were only sparingly identified at the entrance of zoomed-in blood vessels in the brain via confocal microscopy of brain cross-sections (Fig. 4A). In contrast, magnitudes-greater IL-coated PLGA NPs were observed to enter into the brain through the vessel where they initially colocalized to endothelial cells (Fig. 4B), supporting the RBC hitchhiking-BBB shear theory. However, further throughout the caudate/putamen, IL-PLGA DiD NPs were able to actively migrate past endothelial cells after vessel entrance, where the majority was selectively & consistently uptaken into the soma of Iba-1 + microglia (Fig. 4C & Fig. 4F,F’). However, to a lesser degree, IL-DiD NPs also co-localized in von Willebrand factor-positive endothelial cells (Fig. 4D–D’). To confirm microglial uptake vs. membrane-only adsorption, virtual cross-sections of Z-stacked images support the notion that DiD signal is located in the intracellular fraction of Iba-1 + cells (Fig. 4E).

**Figure 5:**
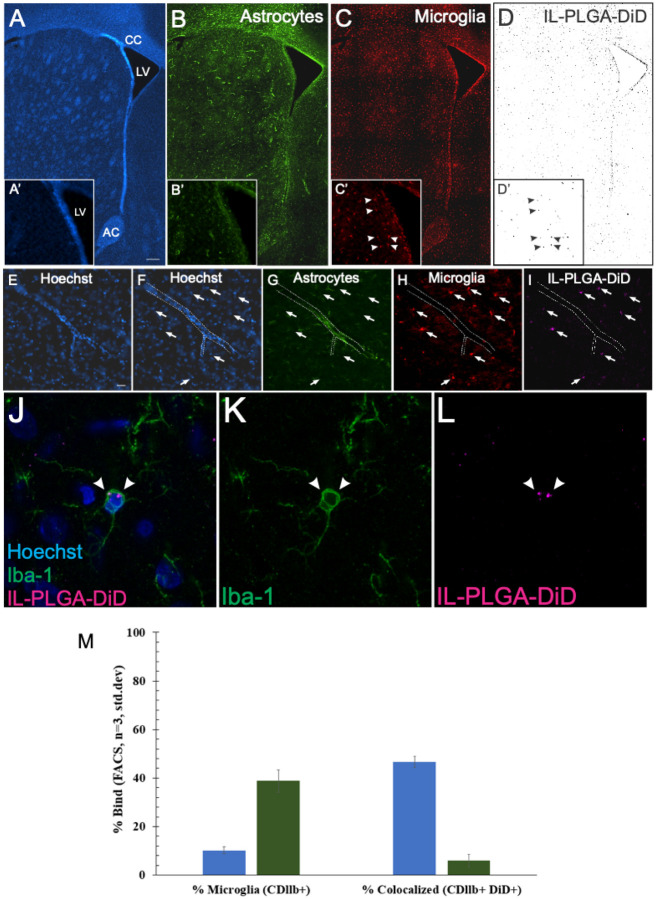
IL-PLGA DiD NPs colocalize selectively with microglia *in vivo*. (A-D) Colocalization of DiD-loaded IL-NPs (D) with microglia (C), but not astrocytes (B), is demonstrated in the parenchyma of the dorsal striatum (inset shows the head of the caudate across panels). (E-I) A blood vessel (outlined in F-E) reveals DiD (I) colocalization with microglia (H), but not astrocytes (G; arrows localize DiD signal across panels). Somal expression of DiD is evident in microglia (J-L). (M) Fractional gated representation of FACS quantification (CDllb+ vs. CDllb+DiD+) of isolated microglia show high colocalization with IL- PLGA DiD NPs (blue) vs. saline background, which is CDllb+(green) only (n=3 internal repetitions of n=1 brain extract/group ± standard deviation). CC=corpus callosum, LV=lateral ventricle, AC=anterior commissure.

To both qualitatively and quantitatively confirm IL- PLGA NP selectivity for cells comprising the HIV reservoir, brain sections (40 μm) collected from rats used above were co-labeled for protein markers of astrocytes (GFAP; Fig. 5B) and microglia (Iba-1; Fig. 5C) with a Hoechst nuclear counterstain (Fig. 5A). Widefield images (10×) of the caudate/putamen within the dorsal striatum, a region of dense HIV viral load in the human brain^32^, demonstrated an apparent colocalization of DiD signal (Fig. 5D) with Iba-1 (Fig. 5C) in the brain of a rat infused with IL-coated NPs. A cross-section of a blood vessel is seen (Fig. 5E–I; 20×) with the astrocytic component of the blood-brain barrier visualized (Fig. 5G). Surrounding microglia (Fig. 5H) are observed to colocalize with DiD signal (Fig. 5I).

To ensure that spectral bleed-through from the near-infrared channel was not accounting for colocalization, Iba-1 was also assessed with a secondary antibody in the green wavelength of the electromagnetic spectrum and DiD signal was confirmed to co-localized with Iba-1 + cells (Fig. 5J–L). Interestingly, when microglia were extracted, isolated, and purified from whole Sprague-Dawley rat brains (at the 6-hour endpoint) treated with saline or IL-PLGA DiD NPs (n = 1/group with n = 3 internal repetitions/group), fluorescence-activated cell sorting (FACS) indicated 46.5 ± 2.3% of microglia were DiD+ (Fig. 5M and Fig. S5), confirming selective microglial uptake identified by confocal imaging.

## Conclusions

In conclusion, we report a novel and highly-effective strategy for nanoparticle delivery to the brain through the development of bioinspired IL coatings that enable spontaneous hitchhiking onto red blood cells post-injection. In addition to tissue specificity, the IL coating also shows preferential uptake into microglia *in vivo*. We also show the successful encapsulation of the ART, abacavir, and our *in vitro* assays indicate that the drug retains efficacy, the IL-NPs are non-toxic to PBMCs, and the IL coating significantly increases uptake of the NPs into cells. In all, bioinspired IL coatings are a promising new technology that could make delivery of a variety of therapeutics into the brain feasible and effective. Future studies will focus on determining efficacy in larger animal models, such as macaques, and in disease models, as well as safety and immune profiling.
